# Exploring Mental Health during the Initial COVID-19 Lockdown in Mumbai: Serendipity for Some Women

**DOI:** 10.3390/ijerph182312542

**Published:** 2021-11-28

**Authors:** Lisa R. Roberts, Shreeletha Solomon, Solomon J. Renati, Susanne Montgomery

**Affiliations:** 1 School of Nursing, Loma Linda University, Loma Linda, CA 92350, USA; 2 Department of Psychology, Martin Luther Christian University, Shillong 793006, Meghalaya, India; shreelethasolomon@gmail.com; 3 Department of Psychology, Veer Wajekar Arts Science and Commerce College, University of Mumbai, Mumbai 400032, Maharashtra, India; renati.solomon@deakin.edu.au; 4 School of Behavioral Health, Loma Linda University, Loma Linda, CA 92350, USA; smontgomery@llu.edu

**Keywords:** COVID-19, global pandemic, maternal and reproductive health, psychosocial health, India

## Abstract

*Background*: This study explored how low-income women already distressed by reproductive challenges were affected during the initial lockdown conditions of the COVID-19 pandemic in Mumbai, India. *Methods*: Women with reproductive challenges and living in established slums participated in a longitudinal mixed-methods study comparing their mental health over time, at pre-COVID-19 and at one and four-months into India’s COVID-19 lockdown. *Results*: Participants (*n* = 98) who presented with elevated mental health symptoms at baseline had significantly reduced symptoms during the initial lockdown. Improvements were associated with income, socioeconomic status, perceived stress, social support, coping strategies, and life satisfaction. Life satisfaction explained 37% of the variance in mental health change, which was qualitatively linked with greater family time (social support) and less worry about necessities, which were subsidized by the government. *Conclusions*: As the pandemic continues and government support wanes, original mental health issues are likely to resurface and possibly worsen, if unaddressed. Our research points to the health benefits experienced by the poor in India when basic needs are at least partially met with government assistance. Moreover, our findings point to the critical role of social support for women suffering reproductive challenges, who often grieve alone. Future interventions to serve these women should take this into account.

## 1. Introduction

The global COVID-19 pandemic has wreaked havoc on human health, which continues to unfold as the subsequent lockdowns instituted have disrupted day-to-day life, and employment and economic status, with additional devastating consequences to physical and mental health—to which, India is no exception [1]. Cases date back to as early as 30 January 2020, and have increased rapidly and continue to affect daily life in India [2].

In India, the state of Maharashtra has experienced among the highest numbers of COVID-19 cases, with 6.61 million cases reported as of October 2021 (https://www.google.com/search?client=firefox-b-1-d&q=covid-19+maharashtra+tracker, accessed on 27 November 2021). The state also reported 133,000 COVID-related deaths [3] despite strict lockdown measures instituted in mid-March, 2020 [4]. These lockdown measures continued [5] for months with constant uncertainty pertaining to severity and duration [6]. Although the national lockdown ended in May 2020, red zones, or high impact zones, cities such as Mumbai continued with subsequent lockdowns or restrictions [7]. Furthermore, a government-approved study comparing COVID-19 infection rates in slums and non-slum communities in Mumbai found markedly higher rates in the slums (54.1% vs. 16.1% respectively). The density of slum communities (crowded residential areas built of substandard housing and typically lacking infrastructure for clean water, and sewage and waste disposal) renders social distancing nearly impossible and poor availability of facilities for hygiene likely further helps induce this disparity [8].

Like elsewhere in the world, the lockdowns, although designed to prevent the spread of COVID-19, have caused fear of subsequent economic consequences. Some predicted that the lockdowns would trigger the first recession in India in the last 40 years. Devastating consequences of the global pandemic on maternal and child health have also been predicted, due to reductions in essential health services expected to contribute to increased maternal deaths, newborn deaths, and stillbirths [9]. In low- and middle-income country, reductions in maternal and child health services are estimated to range from 9.8 to 39.3% by setting, and hunger-related consequences (due to decreased food availability) are expected to increase between 10 to 50%, and result in increases in mortality and morbidity [10]. In India, with poorer members of society already affected by food insecurity, COVID-19 lockdowns and crop devastation by locusts further compound these problems [11,12].

Women of reproductive age are even more vulnerable due to disruption of contraception and other reproductive care services, resulting in unwanted or unintended pregnancies, sexual abuse, and domestic violence, in addition to gender-based economic discrimination (such as lost wages) during lockdown conditions [13,14,15]. Moreover, child marriages in India have increased during the pandemic [16].

Due to the relentless impact of the pandemic, including the imposed lockdowns put in place by India’s government until summer/fall of 2020, experts are increasingly beginning to consider the potential mental health consequences of COVID-19, an issue already discussed as a significant challenge in many affected countries [17,18,19,20,21,22,23]. Complicating this matter further is the dearth of mental health professionals in India, and longstanding stigmatization of mental health [1,24]. In fact, negative attitudes towards mental health professionals in urban India were found to be most prevalent among young adults with lower education and strong religious beliefs—particularly among Hindus and Muslims [25]. Current reports of suicide and increased psychological distress during the pandemic have prompted recommendations for tele-mental health care, social media promotion of preventive measures, and dissemination of reliable information updates pertaining to the virus [1,14,23]. Most expect that the psychological distress present prior to the pandemic will significantly worsen in light of the increasing fear of contracting the virus, in addition to the multiple stressors related to the lockdown [1,19,21]. As a result of the importance placed on women’s childbearing in India’s traditionally patriarchal, pronatalist society, the loss of status, in addition to grief, puts women who have reproductive challenges (trouble conceiving and/or perinatal loss) at high risk for mental health sequelae, thus making them an especially vulnerable sub-group [26,27]. 

As part of a larger study exploring maternal mental health in slum-dwelling women with reproductive challenges in Mumbai, we assessed these women’s mental health using validated scales just prior to the COVID-19 pandemic. We built on this assessment to monitor our participant’s mental health via follow-up phone interviews during the initial COVID-19 lockdown. Using the conceptual framework of the transactional model of stress and coping [28], we explored how this vulnerable group coped in the initial wave of the COVID-19 epidemic. The framework suggests that when stressors (infertility) impact personal goals (such as reproduction), anxiety and distress are likely. When one has little control over the situation, coping efforts are used to try to manage stress. Emotional regulation is the most adaptive coping strategy, but may be thwarted by pressure to meet social expectations, overwhelming obstacles, and lack of social support [29]. However, meaning-based coping, including positive reappraisal, is possible when events (such as subsequent pregnancy) or revised goals (e.g., personal or career growth) may result in emotional well-being, improved functional status, and health behaviors—in a word, resilience [28,30].

Although we expected COVID-related stress to strongly impact these vulnerable women’s lives, we also need to note that the Indian government made the decision to try to balance their strict lockdown with a variety of measures to support those expected to shelter in place [31,32]. In the first three months, these included a government contribution to employee wages for those in the formal sector; collateral-free loans (up to INR 200,000) for self-help groups; food subsidies (5 kg of wheat and rice, 1 kg of legumes per household); provision of cooking gas cylinders for women below the poverty line; stipends specifically for senior citizens, widows, and people with disabilities (INR 1000); support for farmers; relief for daily wage workers; and insurance for medical workers [33,34,35,36,37,38,39]. The initial INR 1.7 trillion (USD 22 billion) three-month relief package was followed by additional relief packages [39], although the exact nature was less clearly documented and declined over time. 

The purpose of this study was to longitudinally explore how these already distressed (by reproductive challenges), low-income women, were affected by ongoing pandemic conditions up to five months into the COVID-19 lockdown. In line with early studies on COVID-19 demonstrating increased mental health symptomology [23,40] and specifically, increased risk of maternal depression and anxiety [41], we hypothesized similar negative health effects in our sample of vulnerable women. However, although the government provided aid during the early lockdown months, we wanted to also explore how this aid, while meager, may have provided an unexpected temporary security and how this may have affected overall mental health and functioning of our participants, despite the pandemic stress.

## 2. Material and Methods

### 2.1. Overview

As part of the larger maternal health mixed-method study, we administered baseline surveys as in-person structured interviews (due to limited literacy and research exposure) with 334 slum-dwelling women of reproductive age, to compare women with reproductive challenges to women without such challenges. The ultimate purpose of the original study was to prepare for a wellness intervention for women experiencing high levels of reproductive related distress. 

In anticipation of a future intervention, we collected interested women’s phone contact information and their consent for us to contact them. Although the COVID-19 pandemic delayed the intervention, in the current sub-study we recruited 98 women from the original group of women with reproductive challenges (stillbirth and perinatal death), who agreed to participate in phone follow-up interviews that focused on life during COVID-19. The first follow-up phone interview was conducted 5 weeks into the lockdown (mid-April 2020), and the second follow-up phone interview occurred approximately five months after the baseline data collection (August 2020, four months into the COVID-19 lockdown). Data was collected by gender- and language-matched, trained local interviewers, teamed up with Accredited Social Health Activists (ASHA) workers who worked with our team to recruit participants. ASHA workers are government supported, trusted women in their communities, who serve the health needs of low-income communities, similar to Community Health Workers (CHWs) in other countries [42]. All interviews were conducted in either Hindi or Marathi, the predominant local languages. 

To best describe the complexity of our participant’s lives, strengthen our interpretations, and further our understanding of the phenomenon of the women’s challenges, we also collected focus group data from the ASHA workers (*n* = 7) and asked several open-ended questions during the interview. 

#### 2.1.1. Qualitative Methods 

Using purposive sampling we conducted a qualitative focus group with seven ASHA workers who live and work in the same community (the Turbhe area of Mumbai) as our participants. Focus groups were conducted by a gender- and language-matched trained facilitator. Participants ranged in age from 26 to 45 (mean age 36) and were all Hindu women. We used a semi-structured guide with questions aligned to our theoretical model (social expectations for women of reproductive age, childbearing concerns, and challenges/stress in the community), and audio-recorded, transcribed, and analyzed the resulting transcriptions using standard methods including coding for emergent themes [43]. We also transcribed any answers to the open-ended questions embedded in the follow-up phone interviews and analyzed them using the same methods.

#### 2.1.2. Quantitative Methods

The baseline survey was informed by prior literature and ASHA worker input from the focus groups and included demographic questions and questions about social support, religious coping, coping style, autonomy, mental health, satisfaction with life, perinatal grief, and post-natal depression. Of the original 334 women, we attempted to contact the 118 women who had indicated that they were interested in future interventions. We were able to contact 98 women (83% response rate) who completed the follow-up phone interviews and are part of the current study. In these follow-up interviews we repeated the mental health questions from the baseline interview and also asked about stress, COVID-19 related issues, and resilience. Ethics Committee approval from the Indian authors’ institutions and Institutional Review Board (IRB) approval from the US authors’ institution was received for the parent study and current study (interviews and focus group), in accordance with the declaration of Helsinki, before commencing data collection. Written informed consent was obtained from participants prior to baseline assessment and verbal consent was obtained prior to follow-up phone interviews. Interview participants were different from focus group participants, but recruited from the same population.

### 2.2. Measures

#### 2.2.1. Descriptive Variables

Demographic variables included age, marital status, religion, education, occupation, and socioeconomic status. Additional descriptive variables included general health status and reproductive history. New descriptive variables from the phone interview included Likert-type, multiple answer, and open-ended questions pertaining to COVID-19 in their communities and how it was affecting their lives. 

#### 2.2.2. Validated Scales

*Hopkins Symptoms Check List–10 (HSCL-10).* An Urdu translation of the HSCL-10 has performed well among a sample of poor Pakistanis [44]. A Hindi translation was used with a poor, rural population in India and found to have good reliability, with a Cronbach’s alpha of 0.84 [45]. Therefore, it was chosen for use among this population who share some characteristics. The measure consists of 10 items, which are rated on a Likert-type scale ranging from (1) not at all, to (4) extremely, with higher scores representing more symptoms of anxiety and depression. Like Syed et al. [44], we used a mean cut-off score of 1.65 or greater to indicate the presence of notable mental health symptoms (anxiety and depression). The Cronbach’s *α* = 0.87 in this study. 

*Perceived Stress Scale–4 (PSS-4).* The PSS-4 is a self-report measure of participants’ global feelings of stress over the last month, and is a shortened version of the Cohen’s 14-item Perceived Stress Scale [46]. The measure asks participants to rate how often they have experienced stress with response options of 0 (never) to 4 (very often). Before summing the four items, positively worded items are reversed so that higher scores indicate higher stress. The PSS-4 is a reliable measure of perceived stress (Cronbach’s *α* = 0.77) [47] developed for research with community samples [48]. The scale is available in many languages and has been used in a variety of populations [47,49,50,51]. In the current sample the Cronbach’s *α* = 0.68.

*Connor Davidson Resilience–10 (CD-RISC 10).* The CD-RISC 10 is a validated shortened version of the original 25-item scale measuring resilience [52,53] among varied study populations [54,55,56,57]. Using a 5-point Likert-type scale of 0 (not true at all) to 4 (true nearly all the time), participants self-report how they have responded to situations. Cronbach’s *α* = 0.85 in this study sample.

Additional validated scales serving as baseline measures in the larger study included the Perinatal Grief Scale [58], Edinburgh Postnatal Depression Scale [59], Social Provision Scale [60], Brief RCOPE (religious coping) [61], shortened Ways of Coping revised scale (emotional/wishful thinking vs. practical coping) [62], Satisfaction with Life Scale [63], and an author-developed autonomy scale as reported elsewhere [26,64]. 

### 2.3. Analytic Quantitative Methods 

Descriptive analyses were undertaken to compare our women in the parent study sample (*n* = 236) with the women who agreed to the phone sub-study presented here (*n* = 98). We then conducted chi square and *t*-tests to determine significant differences between these two groups for the variables of interest. Among the 98 women who agreed to participate in the sub-study, 73% (*n* = 71) participated in a second follow up phone interview four months into the lockdown (5 months post baseline survey) and we used the chi-square or paired *t*-test to explore differences over time in COVID-related variables (which we did not have at baseline). One-way repeated-measures ANOVA was used to longitudinally explore our mental health (HSCL) variable over time (baseline (before COVID-19), and at two follow-ups during COVID-19). We created a mental health change variable by calculating change between the baseline HSCL and the first phone follow-up HSCL. To optimize power and for model-building purposes, we bi-variably explored (using Pearson’s correlations) the independent variables that were significantly associated with mental health (HSCL-10 change). We then included only these variables in the multivariate analyses.

### 2.4. Education and Resources

Women who participated in the phone follow-ups were offered COVID-19-related education and resources, according to their interest and needs. Educational options included guidelines for social distancing and hygiene, in addition to recommendations to maintain or optimize wellbeing during confinement. Additionally, referral resources were available for any women with a high HSCL score or indications of experiencing serious tension or conflict.

## 3. Results

### 3.1. Qualitative Findings

Findings relevant to the current study, summarized by emerging themes from the focus group (FG) discussion and the open-ended follow up questions, helped us understand the significant pressures of the familial expectations on our women in the context of reproductive and work expectations, and how these result in distress. We learned that, although these pressures did not change per se, having the women surrounded by their families during the lockdown (all had to be home, whereas many would usually be out working or seeking work) resulted in unexpected supports that seemed to help the women. This was confirmed by the quantitative results in terms of improved mental health.

The ASHA worker respondents who served as expert consultants about their community of women (*n* = 7) confirmed that most women in their communities are married by 17–18 years of age, and that they are initially housewives who live with their husbands or in a joint family (husband and his parents and siblings). Financial issues may force a young woman to work outside the home, but if that is the case, she is still expected to maintain her household duties. Participants expressed the social expectation that women begin having children soon after marriage. As stated by one ASHA worker, *“She is expected to get pregnant or have a baby compulsorily within a year of marriage”*, which the rest of the FG unanimously and strongly affirmed. Participants further noted that reproductive expectations do not change even if the woman is working outside the home, which often involves manual labor.

*“If women don’t get pregnant even after two to three years of waiting, then the husband will marry again for the second time. Both the wives live together with the man.”*

Though this was stated matter-of-factly, the FG participants went on to discuss the social consequences of reproductive challenges such as lower social status, displacement, abuse, divorce, or abandonment. Furthermore, the ASHA workers indicated that the women are blamed for reproductive challenges, whether they are infertility, miscarriage, stillbirth, or infant death. “*Mother-in-laws constantly taunt—blame women saying ‘you killed the child’...*” even if a medical report indicates otherwise. Some families do offer emotional support to the mother immediately after stillbirth, but nevertheless expect the women to get pregnant again right away. In general, the participants said that the women in their community face enormous family pressure to reproduce and ignore family planning advice from medical professionals or ASHA workers. “*Some women has five months old child but three months pregnant already because of family pressure*”. Sometimes this was felt to be because “*They are not happy with girl child alone*”, alluding to son preference. This turned to frank discussion regarding the prevalence of son preference in their communities. As one participant stated “*There is family pressure to have baby boy.*”; and another comparing the loss of female or male babies said “*They grieve more for boy. They are more disturbed with still born baby boy compared to baby girl*”.

The societal expectations for reproduction (and family pressure, which in some cases was noted to include coercion) combined with social consequences (demoted to lower status within the household, blame, etc.) was noted to add to women’s distress when experiencing reproductive challenges. The ASHA workers noted distress to include grief, fear, crying, intense distress, withdrawal, isolation, and trauma. Participants discussed the intensity and length of grief as variable, and remedies for reproductive challenges were sought at great financial burden to the family with varying results; however, they concluded that the only true way to resolve the distress was to successfully reproduce.

“*Until they get pregnant for the second time. (Family member’s torture and neighbors’ pressure complex [exacerbates] and makes their grief last longer.)*”

These three themes (reproductive expectations, consequences of reproductive challenges, and distress related to reproductive challenges) are somewhat overlapping and compounding. FG participants identified the mental health sequelae of reproductive challenges as an array of emotional distress and symptomology. 

In response to an open-ended question on the phone interviews regarding any changes noted in their relationships during lockdown, women (*n* = 98) noted feeling better supported by their families with everyone together at home during the lockdown. Participants noted that spending more time together resulted in talking more and caring more for each other, which had a positive effect on their relationships.

“*Before my husband use to stay at work and used to spend less time with us, but now he spends much more time with us so things are much better*.”

“*As we are spending much time together our bond has become more strong*.”

A few participants were or became pregnant during the lockdown and noted that they received extra consideration and caring, making them feel special, “Due to my pregnancy my family members have started loving me more.”

In addition to meditation, prayer/worship, maintaining a healthy diet, exercise, and using time productively, an open-ended question on the phone interviews asked the women what else they were doing to try to stay healthy during lockdown. Many of the women again focused on their families, noting that doing household work, as a way of caring for their families and staying busy, had a positive influence on them; in other words, household work was actually perceived as self-care. Others noted extra time for sleep, sewing, and engaging in leisure activities, “playing indoor games”, all things that few would have been able to do before the lockdown as “hustling” for work or survival funds is an expected part of life for these low-income families.

### 3.2. Quantitative Results

#### 3.2.1. Participants

Table 1 and Table 2 compare our overall group of slum-dwelling female participants with those who agreed to participate in the current sub-study with phone follow-ups. The larger study sample (*n* = 334) consisted of women 18 to 42 years old, residing in established slums of Mumbai. More than half (56.9%) lived in a joint family context rather than a nuclear family (42.1%), and identified themselves as daughter-in-law or wife (98.3%), having been married an average of 8.22 years (*SD* 5.82). Most were Hindu (53.4%) women, followed by Muslim (33.9%), Buddhist (9.3%), and other (3.4%) including Christians and Banjaris. Most had low education levels (only 19.5% having higher secondary education or above), and worked as unskilled workers or homemakers (89.4%), the majority had a monthly family income of less than INR 20,714 (approximately USD 274), and considered their families as lower middle class. In general, they deemed themselves as physically and mentally healthy (75.4% and 80.6% indicating no problems, respectively).

The current study sample only included (*n* = 98) women that had indicated the desire to engage in a future self-care intervention based on their own assessment of need, and was compared to the larger study sample, with significant differences noted in terms of occupation, contraceptive use, health, and psychosocial problems. The current sample included more semi-skilled or semi-professional workers; women were more likely to have anemia or other health problems; were more likely to have anxiety, depression, or experience domestic violence; and were more likely to be using some form of contraception. Additionally, the women participating in the phone interviews were significantly more likely to indicate less social support, employ more wishful thinking as a coping style, and have greater autonomy, higher mental distress, and lower satisfaction with life, compared to the larger sample of the parent study (see Table 2).

Table 3 describes changes over time due to the COVID-19 lockdown. At the first follow-up phone contact (5 weeks after COVID-19 lockdown began), participants noted multiple sources for COVID-19 information and updates, relying most heavily on news media (80.6%), with fairly high trust regarding the information received (*M* = 3.62, SD 0.92 on a 1–5 scale). The vast majority indicated that there were no cases in their community. At the second phone follow up (five months after baseline or four months after the initial COVID-19 lockdown), nearly 60% still did not know of any positive cases in their community, although friends and family members were identified as having COVID-19. Most participants lived in one to two room houses in crowded slum neighborhoods. The number of people living in the household (*M* = 5.64, *SD* 2.71) indicated tight living quarters, and less than half (42.9%) indicated the ability to maintain social distancing when allowed outside. On a scale of 1–5, most participants were initially quite worried about becoming infected (*M* = 3.64, *SD* 1.30), and that had not significantly changed by the time of the second phone interview (*M* = 3.55, *SD* 0.88). Lockdown conditions negatively affected monthly family income and food availability, but both improved at the second phone follow up (*p* < 0.004 and *p* < *0*.001 respectively). Obtaining necessitates was negatively affected but did not significantly change over time. In the first phone interview, most women (74.5%) did not note increased tension/conflict in family relationships.

However, by the second follow-up, significantly more respondents noted increased tension or conflict with their children. Initial positive changes related to spending more time together were steady over time. In response to an open-ended question, positive changes were characterized as creating stronger bonds, having their husband home and interacting with their children more, and enjoying leisure activities together. A few participants were pregnant (7.1%) at the outset of the pandemic, and one participant noted, “*Due to my pregnancy my family members have started loving me more*”. In response to a question about what the women were doing to stay healthy during this time, most noted engaging in prayer/worship, some were engaged in some type of physical activity or exercise, and a few noted the importance of a healthy diet, which had significantly increased between the first and second phone interview. Various other activities engaged in were sewing, cooking, doing handy work, caring for children, housework, playing indoor games, and sleep. 

We also analyzed change in mental health variables as the lockdown continued. Perceived stress (PSS-4) and mental health symptoms (HSCL-10) remained stable between the first and second follow-up phone interviews, whereas resilience significantly increased (see Table 3).

#### 3.2.2. Paired Sample *t*-Test of Mental Health 

The current sample’s HSCL at baseline (pre-COVID) was significantly higher (*M* = 2.18, *SD* 0.86) than at the time of the first phone follow-up (*M* = 1.43, *SD* 0.46), *t* (92) = 8.53, *p* < 0.001, *r* = 0.66. To further understand the improvements in mental health during the COVID-19 lockdown period, we then carried out further analysis. 

#### 3.2.3. One-Way Repeated-Measures ANOVA Exploring Mental Health (HSCL) 

To longitudinally assess changes in mental health over five months, we conducted a one-way repeated-measures ANOVA comparing the HSCL scores of participants at three different times: baseline, first phone follow-up, and second phone follow-up. A significant effect was found (*F* (2132) = 28.24, *p* < 0.001). Post hoc analysis conducted using protected *t*-tests revealed that scores decreased significantly from baseline HSCL to first phone follow-up HSCL (*M* = 0.68, *SD* 0.86), and although no significant difference existed from first phone follow-up to second phone follow-up (*M* = −0.45, *SD* 0.55), a significant decrease in HSCL was maintained from baseline to second phone follow-up (*M* = 0.53, *SD* 0.91).

#### 3.2.4. Bivariate Analysis of Mental Health Change over Time

We calculated mental health change as the change from baseline HSCL to phone first follow-up HSCL (HSCL-change). We then explored independent variables associated with HSCL change using bivariate analysis. Variables significantly associated with HSCL-change included perceived stress (−0.272, *p* = 0.011), family income and class (0.277, *p* = 0.034; 0.242, *p* = 0.028 respectively), social support (−0.353, *p* = 0.001), wishful thinking coping style (0.213, *p* = 0.046), and satisfaction with life (−0.487, *p* = 0.000).

#### 3.2.5. Linear Regression Analysis of Predictors of Mental Health Change

Multivariate analysis included significant bivariates of HSCL change from baseline to first phone interview (perceived stress, family income and class, social support, wishful thinking, and life satisfaction), which explained 42% of the variation in mental health change. Only social support and life satisfaction remained significant, and perceived stress was not statistically significant (*p* = 0.053). See Table 4 for a summary of the regression analysis. The post hoc achieved power was greater than 0.80; therefore, there is sufficient statistical power to support the analysis results. 

## 4. Discussion

This study examined changes in mental health among slum-dwelling women in Mumbai during the first four months of India’s pandemic lockdown. These vulnerable, hard-to-reach women were already grieving perinatal losses and at high risk for mental health sequelae due to reproductive challenges, and we wanted to explore how the stress of COVID-19 affected them during these early months of the lockdown. Indeed, the women had indicated their interest in a wellness intervention by sharing their phone contacts with us. Because our sample of women were poor (living in slums), we wanted to also explore if the government aid, although meager, may have provided unexpected temporary security.

Qualitative exploration yielded themes including reproductive expectations, consequences of reproductive challenges, and distress related to reproductive challenges. Reproductive expectations included early childbearing and the importance of producing offspring even if working outside the home. The consequences of reproductive challenges were described in terms of family upheaval and change in social status. These findings are consistent with the literature pertaining to India’s pronatalist, patriarchal traditions [16,27]. Not only were emotional distress and mental health symptomology following perinatal loss vividly described, but these aligned with prior literature and emerging concerns related to maternal mental health during the pandemic [13,27,41]. These findings also validated our quantitative results, further elaborating the complex challenges our participants experienced. This may in part explain the unexpected finding that women’s mental health did not deteriorate during the early COVID lockdown months, as it allowed for their husband’s (and other family members’) presence as a source of additional social support.

The work undertaken by slum communities usually entails working in the informal sector, including manual labor. These meager opportunities came to an abrupt halt with the lockdowns imposed by the Indian government in an effort to stop the rapid spread of the COVID-19 pandemic; therefore, slum-dwelling families were home together. To offset the economic peril of these draconian measures (which included strict shelter in place orders, with even shopping for food regulated and only allowed on certain days, according to one’s residential area), the government offered subsidies and rations. Living in tight quarters, most of the women in our sample admitted they were unable to maintain social distancing and noted significantly more positive cases within their communities over time. Although these poor vulnerable women trusted the pandemic information received, and may have understood the necessity of lockdowns, they found the protections offered lacking, as noted in their responses to the negative effects on their monthly family income, availability of goods, and food. We therefore speculated that these circumstances would increase mental health symptomology among these already at-risk women, which we explored quantitatively.

Surprisingly, compared to the international literature that abounds with examples of poor mental health in persons asked to shelter in place due to COVID-19 prevention measures [17,18,19,20,21,22], women in our study experienced significant mental health *improvements*; in other words, they actually derived mental health benefits during the lockdown. We had originally planned to deliver a wellness intervention to address our participant’s fertility-related mental health challenges. However, before the intervention could be offered, the world was struck with the unprecedented COVID-19 pandemic, and a natural experiment continues to unfold amid the pandemic and associated lockdowns. To better understand these unexpected results, we turned to the literature and simultaneously checked our interpretation of results with our community stakeholder partners, the ASHA workers.

For these Mumbai slum-dwelling, low-income women, having the family together and having everyone stay at home provided an opportunity for quality time, deepening connections, and drawing social support from each other. At baseline, their average HSCL score (2.18) was well above the cut-off score of 1.65, and presents a strong argument for the need of our originally planned wellness intervention. However, within the first month of lockdown, the average HSCL score significantly reduced to 1.43—now within the normal range—and remained within the normal range at the time of the second phone follow-up. The relatively large effect size, repeated measures, and congruence of participants’ short qualitative responses to the phone interviews increases our confidence that the improved HSCL scores are not an artifact. Further supporting these findings, resilience scores also significantly increased during the lockdown between the first and second phone interviews (*M* = 21.48, *SD* 5.33 and *M* = 23.76, *SD* 6.08 respectively, *p* = 0.01).

The women in this study live in established slums, which are close-knit communities that, for many, provide a sense of social support [65]. However, it should be noted that at baseline women in the current sample indicated that they had low social support, which was significantly associated with HSCL in our regression analysis. Indeed, the COVID-19-related lockdown seemed to have ameliorated their perception of low social support. Their positive responses in the phone interviews are aligned with social media research among the general Indian population during lockdown [66]. Prior studies have shown social support to be paramount to the mental health of women dealing with stillbirth, infant death, or infertility [67,68,69]. As the lockdowns continued, our participants noted that they appreciated time with family members (and qualitatively reported that this was particularly true for the presence of their husbands), with nearly as many noting positive changes in relationships as those noting increased tension or conflict. When conflict was noted, it was characterized as not very serious, and did not significantly increase over time (*p* = 0.359).

Our results provide a snapshot in time about the mental health of these vulnerable women early in the pandemic. Clearly, for low-income women in a society that requires daily struggles to eat and survive, the unexpected subsidies and stipends provided a regular source of support they never previously received, which, in conjunction with having their families near them, mitigated the stress of the pandemic for them—at least in the early months of the pandemic. We do not anticipate these positive effects to continue and do not have the ability for further study with the women. As the COVID-19 pandemic has continued much longer than anyone expected, government subsidies were not enough to meet demand and could not continue indefinitely [70]. There will likely be more mouths to feed due to interrupted family planning services [14] and as lockdown restrictions are progressively eased, there may or may not be jobs to return to [2]. These women and their families are mostly part of the informal work sector comprising 90% of India’s working population, and will be disproportionally at risk for this high unemployment, which, without a continuing safety net, will result in dire outcomes, including hunger and loss of housing. As a result, they are progressively prone to resultant health disparities the longer the economic consequences of the pandemic continue [2,71]. 

It is clear that additional work should be done to explore the long-term mental health consequences for the most vulnerable in India’s society. However, as our data covering the first four months of the lockdown show, despite the many negative aspects of the pandemic, our participants reportedly have consistently employed strategies to stay healthy through meditation, prayer or worship, productive use of time, and exercise, pointing to a strong innate resilience that was functional with very little investment by the government. Even eating a healthy diet significantly increased (*p* = 0.009), perhaps in response to the education provided at the end of the first phone interview, in addition to making use of government rations, which included legumes, rice, and grain [38]. The return to basic staples may have resulted in a healthier diet than that eaten pre-pandemic [72]. Furthermore, employing our educational options to optimize wellbeing in confinement (suggested self-help strategies for physical and mental health) seems to have been effective for them in handling the situation. At the end of the study, five months after baseline measures and four months into lockdown, their perceived stress scores were in the low range and had not increased over time (*p* = 0.922), initial improvement in mental health symptomology (HSCL) was maintained, and resilience increased. Again, we do not expect these positive trends to continue, especially as the financial stipends decreased when the pandemic lingered for longer than anticipated. However, this natural experiment showed that if slum-dwelling families have even a small amount of governmental support (however modest), which currently does not occur in India, health overall increases despite a stressful life-threatening pandemic. 

While reporting results of this novel study, we acknowledge certain limitations that were unavoidable during lockdown conditions. Although participants consented to participate in the phone interviews, given the privacy limitations of being in tight living quarters with family members constantly present, participants may not have reported negative responses, as much due to fear of being overheard and subsequent retribution. There is also a risk of positive response bias, as participants may have underreported their struggles to maintain social distancing, which is nearly impossible in the context of crowded urban slums [73], although our data seems to reflect this reality quite accurately. Fear of stigma and blame around COVID-19 [70] may have also decreased participants’ disclosure of known contacts within their families and communities. Fear may be amplified by drones being used to monitor lockdown compliance [70] and the looming threat of legal action [5]. Strengths of the paper include the intentional mixed-methods exploratory sequential design, which helped us contextualize the life experiences of our participants. Furthermore, we believe this resulted in our congruent, although unexpected, findings of improved mental health and resilience across multiple quantitative, well-validated measures, and our qualitative data. Although we cannot speak to how this may have changed further into the pandemic, it does indeed show that financial support matters, especially to the poorest in society. It also suggests that any future intervention (post COVID-19 lockdowns) for these women who suffer from poor mental health because of their reproductive challenges should, at least in part, focus on helping them find social support to address the isolation many experience because of the deeply shameful experience of not being able to bear children.

Given the few mental health research papers published pertaining to the global pandemic, particularly among poor populations in India [66], this paper adds importantly to the literature. The longitudinal design provides a glimpse of unexpected positive change in the midst of the COVID-19 crisis. Follow-up studies and further analyses are needed as the situation and aftermath continue to unfold. 

## 5. Conclusions

Slum-dwelling women in Mumbai reported experiencing increased social support during the first four months of the national lockdown. For the present time, in combination with initial governmental measures of support during the lockdown, this support is associated with an improvement in the mental health distress they experience due to reproductive challenges. In a traditional, pronatalist context in which women’s status is directly affected by producing children—particularly sons—reproductive challenges often result in significant mental health distress, especially as they are often judged by their family members about their reproductive capacity. The finding that positive changes in mental health were found in our participants suggests that, although forced, the increased family cohabitation improved family relationships and social support. Future research should also explore the degree to which the positive changes in mental health noted in the current study may be transient, and particularly to determine if these were solely tied to the unexpected financial support the families received from the government, or if the positive results had more to do with relatively few cases originally noted in the social environment of the women—which surely would have increased exponentially over time. Once the supportive measures are removed after the lockdown, and their lives are likely strongly impacted by either COVID-19 or the resulting economic downturn, we expect their original mental health challenges will resurface. With health systems already ill equipped to provide mental health services in this context, we are likely to see worsening mental health among those with mental health issues pre-COVID, suggesting the need for community-based non-stigmatizing wellness programs that will aid these vulnerable women to deal with past and emerging stresses. 

## Figures and Tables

**Table 1:** Comparison of demographics of phone survey sub-group with all other participants.

Characteristic		Phone-Survey Participants *n* = 98	All Other Participants *n* = 236
		Frequency *n* (%)	Frequency *n* (%)
Household position *n* = 334	Wife	58 (59.2)	139 (58.9)
	Daughter-n-law	39 (39.8)	93 (39.4)
	Head of household	0	2 (0.8)
	Daughter	1 (1.0)	2 (0.8)
Religion *n* = 334	Hindu	46 (46.9)	126 (53.4)
	Muslim	43 (43.9)	80 (33.9)
	Buddhist	8 (8.2)	22 (9.3)
	Other^a^	1 (1.0)	8 (3.4)
Highest level of education *n* = 334	None (illiterate)	16 (16.3)	43 (18.2)
	Primary	33 (33.7)	82 (34.7)
	Secondary	33 (33.7)	65 (27.5)
	Higher-secondary or graduate	16 (16.3)	46 (19.5)
Current occupation *** *n* = 334	Unskilled worker/Homemaker	72 (73.5)	211 (89.4)
	Semi-skilled worker to semi-professional	26 (26.5)	25 (10.6)
Monthly family income *n* = 326	≤ Rs. 2091–10,356	40 (41.2)	83 (36.2)
	Rs. 10,357–20,714	51 (52.6)	126 (55.0)
	Rs. 20,715–41,430+	6 (6.2)	20 (8.7)
Time to reach a health facility *n* = 334	Less than 15 min	41 (41.8)	97 (41.1)
	15 to 30 min	55 (56.1)	129 (54.7)
	More than 30 min	2 (2.0)	10 (4.2)
Tobacco/paan use *n* = 334	None	87 (88.8)	216 (91.5)
	Tobacco/paan use	11 (11.2)	20 (8.5)
Health problems *** *n* = 334	None	51 (52.0)	178 (75.4)
	Anemia	33 (33.7)	38 (16.1)
	Other^b^	14 (14.3)	20 (8.5)
Psychosocial problems *** *n* = 329	None	54 (55.7)	187 (80.6)
	Anxiety	40 (41.2)	43 (18.5)
	Depression	2 (2.1)	1 (0.4)
	Domestic violence	1 (1.0)	1 (0.4)
Family style *n* = 333	Nuclear	45 (45.9)	99 (42.1)
	Joint	53 (54.1)	136 (57.9)
Contraceptive method used * *n* = 331	None	72 (73.5)	199 (85.4)
	Sterilization	7 (7.1)	16 (6.9)
	Other^c^	19 (19.4)	18 (7.7)
		*M* (SD)	*M* (SD)
Years of age		26.28 (4.73)	26.38 (5.06)
Years married		7.82 (5.06)	8.22 (5.82)
Number of pregnancies		3.05 (1.81)	2.93 (1.71)

Notes: * = *p* < 0.05, *** = *p* < 0.001; Other^a^ = Christian, Banjari; Other^b^ = Thyroid disease, weakness, malaria, hypertension, TB, stones, calcium deficiency; Other^c^ = Medicine, condoms, IUD, surgery.

**Table 2:** Independent sample *t*-tests comparing variables of interest between the phone survey sub-group and all other participants.

Parameter	Phone-Survey Participants *n* = 98	All Other Women *n* = 233	*t* (330)	*p*	95% CI
	*M (SD)*	*M (SD)*			
Social provision of support	37.16 (6.91)	39.01 (5.46)	−2.544	0.011	[−3.26–0.41]
Positive religious coping	4.57 (2.55)	4.36 (2.45)	0.696	0.487	[−0.38–0.79]
Negative religious coping	8.79 (2.08)	8.64 (2.15)	0.571	0.568	[−0.36–0.65]
Overall religiosity	1.45 (0.79)	1.56 (0.88)	−1.148	0.252	[−0.31–0.08]
Wishful thinking	12.04 (4.20)	10.63 (4.13)	2.737	0.007	[0.39– 2.42]
Practical coping	14.30 (3.99)	14.21 (4.33)	0.168	0.867	[−0.91–1.09]
Autonomy	7.99 (1.97)	7.27 (2.00)	2.953	0.003	[0.23–1.19]
Mental health	2.18 (0.86)	1.59 (0.61)	5.956	0.000	[0.39–0.78]
Satisfaction with life	22.01 (9.10)	26.39 (6.62)	−4.319	0.000	[−6.39–−2.38]

**Table 3:** Descriptive data from phone survey detailing changes over time during the COVID-19 lockdown.

Characteristic	1st Phone Survey *n* = 98	2nd Phone Survey *n* = 71	*p* Value
	*M* (SD) or *n* (%)	*M* (SD) or *n* (%)	*X*^2^ or Paired *t*-Test
COVID positive community members:			
None	96 (98.0)	41 (57.7)	0.029
Family member	1 (1.0)	4 (5.6)	0.081
Friend/s	0	3 (4.2)	0.040
Neighbor/s	1 (1.0)	6 (8.5)	0.016
Worry about contracting COVID (range 1–5)	3.67 (1.38)	3.55 (0.88)	0.490
COVID negatively affected:			
Monthly income	88 (89.8)	52 (73.2)	0.004
Obtaining necessities	44 (44.9)	22 (31.0)	0.067
Food availability	49 (50.0)	10 (14.1)	0.001
Birth control	4 (4.1)	0	0.085
PSS-4 (range 0–16)	6.24 (2.63)	6.19 (2.82)	0.922
HSCL-10 (cut-off score ≥1.65)	1.42 (0.48)	1.47 (0.47)	0.514
Increased tension/conflict with:			
Husband	19 (19.4)	18 (25.4)	0.354
In-laws	13 (13.3)	7 (9.9)	0.498
Children	2 (2.0)	7 (9.9)	0.025
Friends	0	0	
Neighbors	0	0	
Seriousness of conflict (range 1–5)	1.49 (0.83)	1.60 (1.03)	0.359
Positive changes in relationships:	22 (22.4)	15 (21.1)	0.837
Currently pregnant			
Yes	7 (7.1)	3 (4.2)	0.440
Unsure	1 (1.0)	1 (1.4)	0.825
Resilience (range 0–40)	21.48 (5.33)	23.76 (6.08)	0.010
Strategies to stay healthy:			
Meditation	7 (7.1)	9 (12.7)	0.225
Prayer/worship	76 (77.6)	55 (77.5)	0.989
Healthy diet	9 (9.2)	17 (23.9)	0.009
Productive use of time	1 (1.0)	1 (1.4)	0.825
Exercise	12 (12.2)	11 (15.5)	0.543
Minutes per day spent exercising	45 (21.21)	30 (0)	0.500
Exercise number of days per week	6 (1.41)	6.25 (1.06)	0.500

**Table 4:** Summary of regression analysis for variables predicting mental health change (HSCL change) assessed by first phone follow-up during the Mumbai COVID-19 lockdown.

Variable	*B*	*SE B*	β
Constant	1.86	0.65	
Income	0.16	0.15	0.10
Class	0.26	0.18	0.14
Social Support	−0.02	0.01	−0.19 *
Coping style-Wishful thinking	0.03	0.02	0.13
Perceived stress	−0.07	0.03	−0.18
Life satisfaction	−0.04	0.01	−0.40 ***
*R* ^2^	0.42		
*F* for change in *R*^2^	9.13 ***		

* *p* < 0.05, *** *p* < 0.001.

## Data Availability

The datasets used and analyzed during the current study are available from the corresponding author on reasonable request.
